# Attack Algorithm for a Keystore-Based Secret Key Generation Method

**DOI:** 10.3390/e21020212

**Published:** 2019-02-23

**Authors:** Seungjae Chae, Young-Sik Kim, Jong-Seon No, Young-Han Kim

**Affiliations:** 1 The Department of Electrical and Computer Engineering, Institute of New Media and Communications (INMC), Seoul National University, Seoul 08826, Korea; 2 The Department of Information and Communication Engineering, Chosun University, Gwangju 61452, Korea; 3 The Department of Electrical and Computer Engineering, University of California, San Diego, La Jolla, CA 92093, USA

**Keywords:** information-theoretically secure, key generation, key management, keystore seed, one-key-for-one-file

## Abstract

A new attack algorithm is proposed for a secure key generation and management method introduced by Yang and Wu. It was previously claimed that the key generation method of Yang and Wu using a keystore seed was information-theoretically secure and could solve the long-term key storage problem in cloud systems, thanks to the huge number of secure keys that the keystone seed can generate. Their key generation method, however, is considered to be broken if an attacker can recover the keystore seed. The proposed attack algorithm in this paper reconstructs the keystore seed of the Yang–Wu key generation method from a small number of collected keys. For example, when t=5 and $l=2^{7}$, it was previously claimed that more than $2^{53}$ secure keys could be generated, but the proposed attack algorithm can reconstruct the keystone seed based on only 84 collected keys. Hence, the Yang–Wu key generation method is not information-theoretically secure when the attacker can gather multiple keys and a critical amount of information about the keystone seed is leaked.

## 1. Introduction

Data storage and transmission have been frequently used in recent public cloud systems. It is important to use secure keys in the cloud system, because users using a password can be vulnerable to dictionary attacks [1]. It is well known that secure keys reveal less user information than the password method. Thus, secure keys have been used in various fields such as file encryption, access to virtual private networks, and user authentication [2]. However, conventional key generation methods have many problems in terms of long-term file management, where each file should be independently encrypted with random secure keys since it has the characteristics of long-term file storage and frequent user access. Otherwise, cloud systems are not secure for ciphertext-only attack or chosen-plaintext attack [3]. To make one-key-for-one-file secure encryption for long-term data protection, a new secure key generation method using the keystore seed was proposed in [4] claiming that their method could make many information-theoretically ϵ-secure keys. In this paper, we propose a new method to break their key generation by reconstructing the keystore seed using a small number of collected keys.

This paper is organized as follows. In Section 2, the secure key generation and management methods are reviewed. In Section 3, we propose an attack algorithm of the information theoretically ϵ-secure key generation method in [4] and show the successful attack probability. In Section 4, we analyze the modified Yang-Wu’s scheme with the hashed key [5], where information is not theoretically ϵ-secure, but has only computational security. Finally, Section 5 concludes this paper.

## 2. Key Generation and Management Based on Keystore Seed

In this section, we briefly explain the secure key generation and management methods in [4].

### 2.1. Key Generation

There is a keystore seed K=K(0)K(1)⋯K(L−1), which is a randomly generated *L*-bit binary sequence, where K(i) is the *i*-th bit of the keystore seed for 0≤i≤L−1. Let $a_{j}$ be a sub-sequence of length *l* of the keystore seed and let $m_{j}$ be a keystore seed index of the first element of $a_{j}$, where $0\leq m_{1}<m_{2}<\ldots <m_{t}\leq L-1$. Then, $a_{j}$ is represented as $a_{j}=K\left(m_{j}\right)K\left(m_{j}+1\right)\cdots K\left(m_{j}+l-1\right)$. The key $k_{i}$ of length *l* is generated as $k_{i}=a_{1}⊕a_{2}⊕\cdots ⊕a_{t}$, where ⊕ denotes the bit-wise exclusive OR. The set of all possible keys generated from the keystore seed *K* is denoted as $\Psi =\{k_{i}|1\leq i\leq \Lambda \}$, where Λ is Lt. This key generation method is expressed as the (L,l,t)-key generation scheme, where *l* is the length of each key and *t* is the number of subkeys of keystore seed for the generation of each key.

### 2.2. Key Management

After key generation, the generated keys can be used in the following way:

1. A file is encrypted using a key $k_{i}$ randomly selected from set Ψ.
2. Attach the key index information $i=(m_{1},m_{2},\cdots ,m_{t})$ into the encrypted file and send it.
3. To decrypt an encrypted file, the encryption key $k_{i}$ is regenerated from the secure stored keystore seed and the received file using $k_{i}$ is decrypted using the attached key index information *i*.

The keystore seed should be protected in a secure memory that cannot be accessed by outside users. Even though the key index information is available, any information on the keystore seed should not be disclosed.

### 2.3. Information-Theoretically ϵ-Secure Keystore

The information-theoretically ϵ-secure for arbitrarily small ϵ is defined according to the following specifications.

**Definition** **1** ([4])**.** *A keystore $\Psi =\{k_{i}\;|\;1\leq i\leq \Lambda \}$ of keys of length l generated from a keystore seed K is said to be information-theoretically ϵ-secure for 0≤ϵ<1, if the properties in the following theorems hold.*

**Theorem** **1** ([4])**.** *For 1≤i≤Λ and arbitrarily small ϵ>0, all keys $k_{i}$ are randomly and uniformly distributed over ${\left\{0,1\right\}}^{l}$ as*

$$\mathrm{Pr}\left\{k_{i}=k_{j}\right\}\leq \left(1-\epsilon \right)\times 2^{-l}+\epsilon .$$

**Theorem** **2** ([4])**.** *For all pairs of independent indices i,j,1≤i,j≤Λ,*

$$H\left(k_{j}|i,j,k_{i}\right)\geq H\left(k_{j}|j\right)\times \left(1-\epsilon \right)=l\left(1-\epsilon \right).$$

Yang and Wu [4] stated that Theorem 2 can be extended to the following argument.

**Argument** **1** (*n*-th order of Theorem 2)**.** *For all independent $i,j_{1},\ldots ,j_{n}$, where $1\leq i,j_{1},\ldots ,j_{n}\leq \Lambda$, we have*

(1) $$\begin{array}{cc} H(k_{i}|j_{1}, & \ldots ,j_{n},i,k_{j_{1}},\ldots ,k_{j_{n}})\geq \\ & H\left(k_{i}|i\right)\times \left(1-\epsilon \right)=l\left(1-\epsilon \right). \end{array}$$

In this paper, we will demonstrate that this argument is only true for a very small *n*.

## 3. Linear Attack on Key Generation and Management

### 3.1. Linear Attack Algorithm

**Figure 1:**
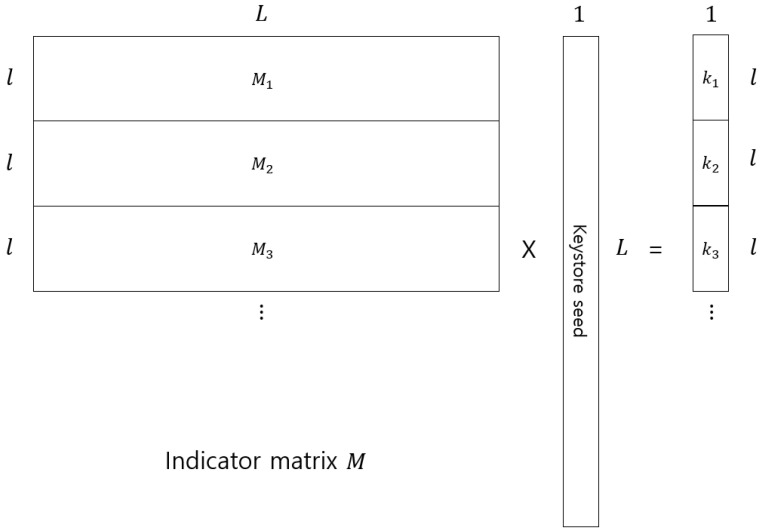
Matrix operation to find keystore seed.

In this section, we propose an attack algorithm to reconstruct a keystore seed from a number of collected keys. For example, assume that we have some keys with t=5 as presented in [4]. Each key has 5 indices and consists of 5 binary exclusive OR subkeys of length *l* starting at given indices. Each key can make l×L submatrix $M_{i}$ shown on the left side of Figure 1. Each $M_{i}$ consists of *l* indicator vectors to generate key $k_{i}$. For example, we have one key with index i=(1,3,4,6,7). Then, the indicator vector $e_{1}^{0}$ is 0101101100⋯00 (All 0 except indices 1,3,4,6,7). Next, the indicator vector $e_{1}^{1}$ is a circular shift to the right of $e_{1}^{0}$. Rows of $M_{i}$ consist of $e_{i}^{0},\cdots ,e_{i}^{l-1}$ and rank$(M_{i})=l$ because it has *l* independent indicator vectors. If the tl<<L condition is not satisfied, there are dependent indicator vectors due to overlap by cyclic shift. The indicator matrix *M* is made by stacking up $M_{i}^{\prime }s$. Consequently, we stack up submatrices until *M* satisfies rank(M)=L. Finally, we find keystore seed using the system of linear equations as Figure 1 because *M* becomes full rank and it is invertible. The attack algorithm is summarized in Algorithm 1. If the indicator matrix *M* has rank *L* by stacking up several indicator submatrices, *Argument 1* is not correct for a sufficiently large *n* to make *M* full rank. Thus, their key generation method is not secure.

**Algorithm 1** Successful attack probability with *R* keys**Input: Variables L,l,R,t** **Output: True if the indicator matrix rank is larger than or equal to***L* **for***i* from 1 to *R***do**  key_index_set← Randomly select *t* integers in range of (0, L−1)  ${e_{i}}^{0}\leftarrow$ indicator vector of key_index_set of length *l*  **for**
*j* from 1 to l−1
**do**   ${e_{i}}^{j}\leftarrow$ circular cyclic shift right once of ${e_{i}}^{j-1}$  **end for**  $M_{i}\leftarrow stack\left\{{e_{i}}^{0},\cdots ,{e_{i}}^{l-1}\right\}$  $M=stack\{M_{1},\cdots ,M_{i}\}$  **if** rank(M)≥L
**then**   return True  **end if** **end for**

Let *Z* be a random variable defined as

$$Z=\left\{\begin{array}{cc} \;1 & \mathrm{if}\;rank(M)=L\\ \;0 & \mathrm{if}\;rank(M)\neq L. \end{array}\right.$$

With this random variable, the left-hand side of (1) can be rewritten as

(2) $$\begin{array}{c} H(k_{i}|j_{1},\cdots ,j_{n},i,k_{j_{1}},\cdots ,k_{j_{n}})=\\ \;H\left(k_{i}|j_{1},\cdots ,j_{n},i,k_{j_{1}},\cdots ,k_{j_{n}},Z=1\right)P\left(Z=1\right)\\ \;+H\left(k_{i}|j_{1},\cdots ,j_{n},i,k_{j_{1}},\cdots ,k_{j_{n}},Z=0\right)P\left(Z=0\right), \end{array}$$

where P(Z=1) means that keystore seed is reconstructed and key’s entropy goes to 0 because $k_{i}$ is automatically determined with key index *i*. Therefore, (2) only contains the P(Z=0) case. Since $H(k_{i}|j_{1},\cdots ,j_{n},i,k_{j_{1}},\cdots ,k_{j_{n}})\leq l$, we have

$$\begin{array}{c} H(k_{i}|j_{1},\cdots ,j_{n},i,k_{j_{1}},\cdots ,k_{j_{n}},Z=0)P\left(Z=0\right)\\ \;\leq lP(Z=0). \end{array}$$

According to numerical analysis, P(Z=0) becomes almost 0 when the number of collected keys increases, which means that the lower bound of entropy in the *n*-th order expansion in *Argument 1* is not correct for a large *n*. Although *Argument 1* is correct for very small *n*, it is not useful in that they could not generate many secure keys because the purpose of their proposed method is to deal with one-key-for-one-file in cloud systems. In other words, when the entropy of the generated keys becomes 0, the keystore seed cannot be used to generate secure keys anymore. Thus, attackers can reconstruct the keystore seed with high probability, which means that their key generation method is no longer information-theoretically ϵ-secure. In the next subsection, we will show the number of collected keys to make rank(M)=L by numerical analysis.

### 3.2. Successful Linear Attack Probability

**Figure 2:**
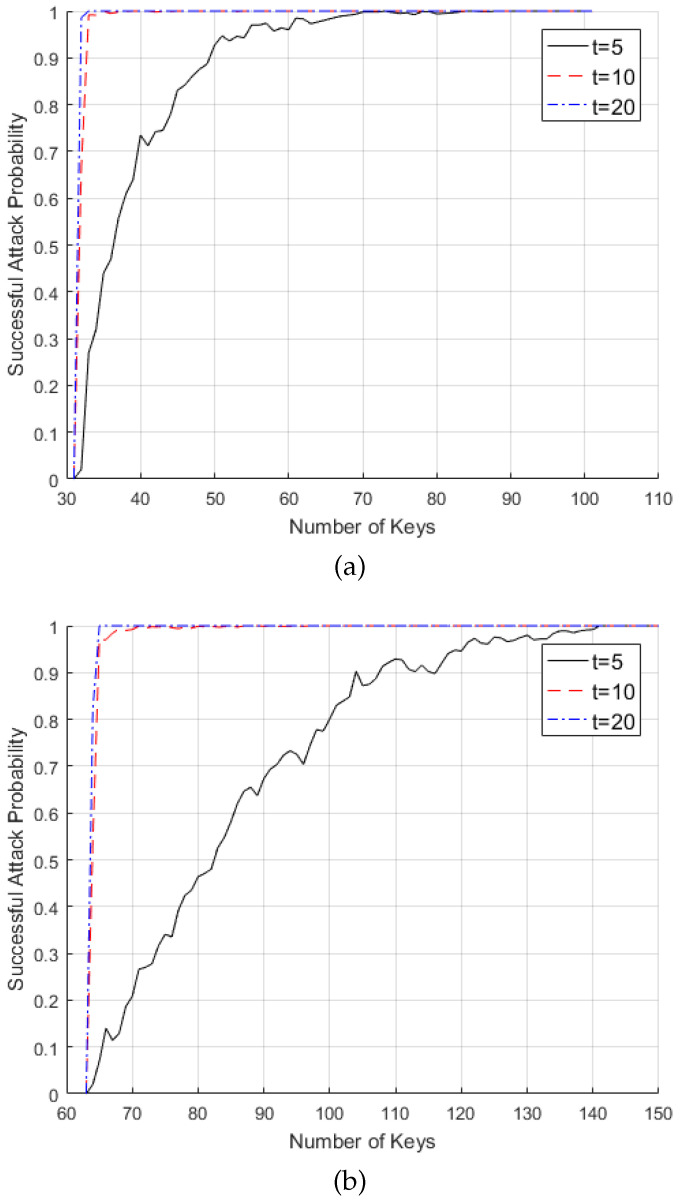
Successful attack probability of the proposed attack algorithm when: (**a**) $L=2^{12}$, $l=2^{7}$, (**b**) $L=2^{14}$, $l=2^{8}$.

The successful attack probability with *R* keys is given as a probability that an indicator matrix *M* has rank larger than or equal to *L* by using *R* keys as in Algorithm 1. Clearly, at least L/l keys are required to make *M* with full rank. Figure 2 shows that the successful attack probability of the key generation algorithm in [4] is numerically derived for $L=2^{12},2^{14},l=2^{7},2^{8}$, respectively, when t=5,10,20. Table 1 lists the successful attack probability in Figure 2 for several numbers of *R*.

## 4. Information Theoretic Weakness of Modified Yang-Wu’s Schemes with Hashed Keys

The forward secrecy is a property such that if a secret key is compromised, past keys are not compromised. According to the key generation method in [4], several keys are generated from one keystore seed through a linear combination. If the number of generated keys is large enough, the newly generated key will have only a very small entropy from previously generated keys. This idea can be checked via the following observation.

For binary independent random variables *X* and *Y*, suppose that H(X)=H(Y)=1 and H(X,Y)=2. Then, we have

$$\begin{array}{cc} H(X,Y|X⊕Y) & =H(X,Y,X⊕Y)-H(X⊕Y)\\ & =H(X,Y)-H(X⊕Y)=1. \end{array}$$

**Figure 3:**
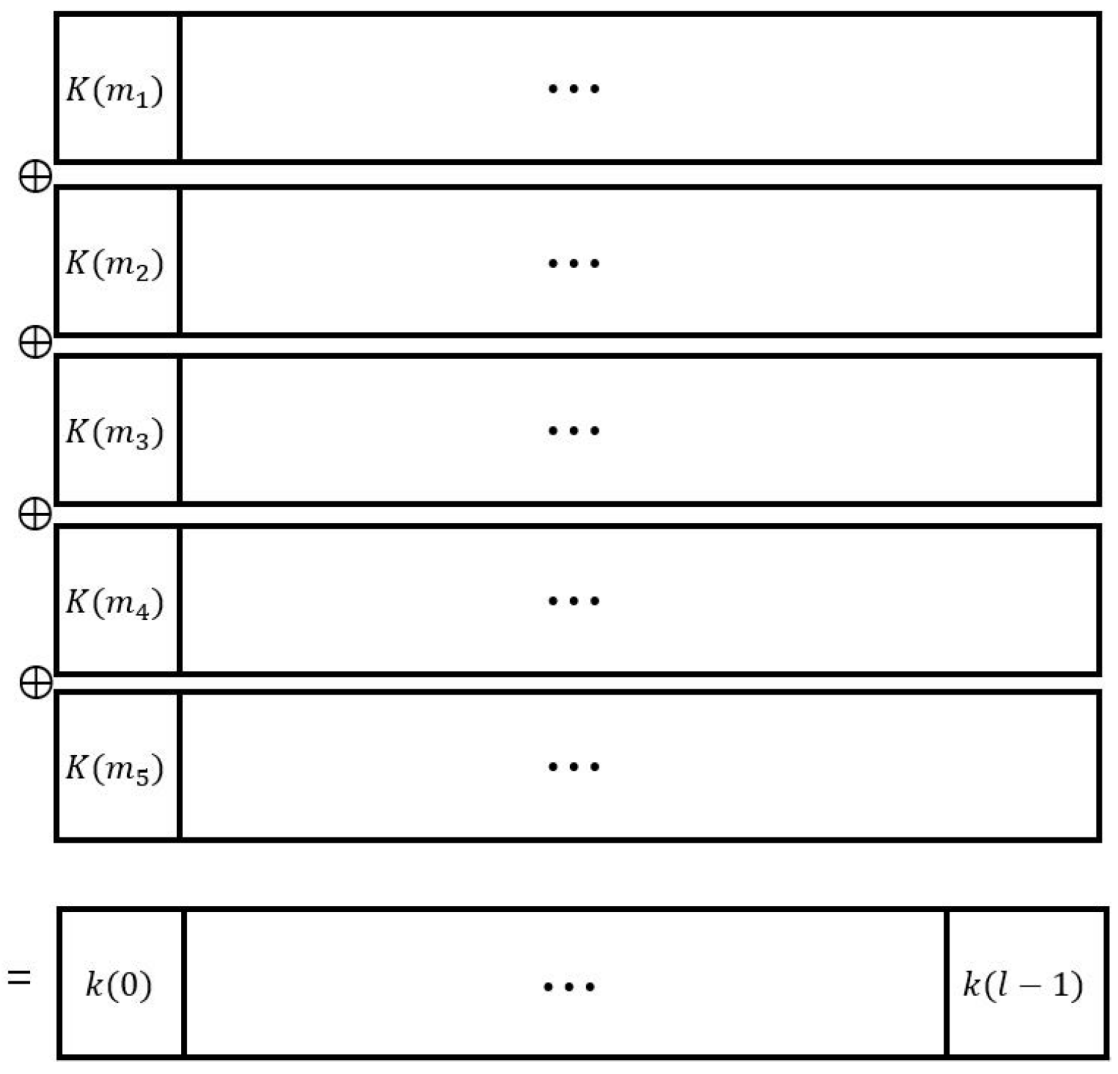
Key generation by subkeys.

This can easily be extended and applied to Yang and Wu’s algorithm intended to provide independent and uncorrelated secret keys for the one-key-for-one-file long-term secure system. Assume that we have one key generated from tl bits of keystore seed as in Figure 3. If we know the subkeys $K\left(m_{j}\right)K\left(m_{j}+1\right)\cdots K\left(m_{j}+l-1\right)$ for j=1,⋯,t−1, we can derive the subkey $K\left(m_{t}\right)K\left(m_{t}+1\right)\cdots K\left(m_{t}+l-1\right)$ since we know the key k(0)k(1)⋯k(l−1). As *t* increases, the number of subkeys generating a key becomes large. This becomes a weak point when giving the indicator matrix *M* a full rank in Section 3. As the simulation results show that the successful attack probability of the proposed attack algorithm for t=10,20 increases abruptly compared to t=5 when the number of collected keys becomes large. In addition, the successful attack probability becomes very large as *t* increases. Therefore, a large value of *t* for the key generation scheme should be avoided.

In real applications, it is very important to provide a way of strong protection for the keystored seed. However, in a cloud environment, there is a possibility that some information can be disclosed during the processing such as key generation, file encryption, or decryption, due to undiscovered weakness of systems or side channel attacks as in [6]. In this paper, we show that it is possible to reconstruct the entire keystore seed even if a very small number of generated keys (i.e., 84 keys) are leaked compared to the total size of the possible keys (i.e., $2^{53}$ keys).

In order to reduce the risk of keystore seed reconstruction, the encryption using a hashed key h(k) was proposed in [5], where *k* is a generated key from the keystore seed and h(·) is a one-way hash function. It is true that encryption with a hashed key could avoid the proposed linear attack of keystore seed reconstruction. However, avoiding the linear attack does not guarantee information-theoretically ϵ-secure since hashed keys are the same number of bits as original keys. If the original keystore is not information-theoretically ϵ-secure, hashed keys are not also information-theoretically ϵ-secure since hashing is one to one mapping. Hashing only increases computational complexity, but it does not guarantee key entropy.

The hashed key can be a countermeasure for the proposed linear attack. Moreover, by introducing a hash chain for key generation, it is possible to increase both the computational complexity of the linear analysis and the number of possible keys. Let us set each subkey as $a_{j}=K\left(m_{j}\right)K\left(m_{j}+1\right)\cdots K\left(m_{j}+l-1\right)$ for j=1,⋯,5. Then, the key $k_{j}$ is generated as $k_{j}=h\left(h\left(h\left(h\left(h\left(a_{1}\right)⊕a_{2}\right)⊕a_{3}\right)⊕a_{4}\right)⊕a_{5}\right)$, where $a_{i}$ is a subkey and h(·) is a one-way hash function from ${\left\{0,1\right\}}^{*}$ to ${\left\{0,1\right\}}^{l}$. Note that if the order of applying $a_{i}$ is changed, the generated key is completely different when a cryptographic hash function such as SHA-2 or SHA-3 is used. Even though this type of countermeasure cannot guarantee information-theoretically ϵ-secure keys, but it can be a cryptographically secure way.

## 5. Conclusions

As the demand for long-term data over the public clouds increases, a large number of secure keys are needed. To deal with this problem, Yang and Wu proposed a new key generation method using the keystore seed [4]. In this paper, we proposed an attack algorithm for their key generation method, where a small number of collected keys can be used to reconstruct the keystore seed with high probability. Although the encryption using a hashed key could avoid the proposed reconstruction attack, it still does not guarantee the information-theoretically ϵ-secure in certain situations where some information is leaked. Therefore, a new secure key generation method with keystore seed can be studied in future research.

## Figures and Tables

**Table 1:** Successful attack probability of the proposed attack algorithm.

$L=2^{12},l=2^{7}$			
Number of keys	t=5	t=10	t=20
R=32	0.02	0.664	0.986
R=40	0.735	1	1
R=84	1	1	1
$L=2^{14},l=2^{8}$			
Number of keys	t=5	t=10	t=20
R=64	0.02	0.540	0.820
R=70	0.208	0.992	1
R=100	0.801	1	1
R=141	1	1	1
